# Shifts in Pediatric Respiratory Virus Trends After the COVID‐19 Pandemic: Insights From a 12‐Year Hospital‐Based Study in South Korea

**DOI:** 10.1155/ijpe/4499218

**Published:** 2026-06-26

**Authors:** Eunkyo Ha, Ju Hee Kim, Jeewon Shin, Won Seok Lee, Eun Lee, Hey Sung Baek, Man Yong Han

**Affiliations:** ^1^ Department of Pediatrics, Kangnam Sacred Heart Hospital, Hallym University College of Medicine, Seoul, South Korea, hallym.ac.kr; ^2^ Department of Pediatrics, Kyung Hee University Medical Center, Kyung Hee University School of Medicine, Seoul, South Korea, khu.ac.kr; ^3^ Department of Pediatrics, CHA Ilsan Medical Center, CHA University School of Medicine, Goyang, South Korea, cha.ac.kr; ^4^ Department of Pediatrics, Chonnam National University Hospital, Chonnam National University Medical School, Gwangju, South Korea, jnu.ac.kr; ^5^ Department of Pediatrics, Kangdong Sacred Heart Hospital, Hallym University College of Medicine, Seoul, South Korea, hallym.ac.kr; ^6^ Department of Pediatrics, CHA Bundang Medical Center, CHA University School of Medicine, Seongnam-si, South Korea, cha.ac.kr

**Keywords:** climate changes, coinfection, COVID-19 pandemic, epidemiological models, respiratory tract infection, seasonal variation

## Abstract

**Background:**

The COVID‐19 pandemic disrupted the seasonal patterns of pediatric respiratory infections, likely due to an immunologic gap. However, the roles of weather conditions and viral interactions in age‐specific transmission, seasonality, and coinfections remain unclear.

**Methods:**

This retrospective study analyzed data from children hospitalized with respiratory tract infections between January 2012 and December 2023 at a single tertiary center in Korea. Six common respiratory viruses were identified via multiplex polymerase chain reaction (PCR). The study period was divided into prepandemic (January 2012 to January 2020), pandemic (February 2020 to November 2021), and postpandemic (December 2021 to December 2023) periods. Pandemic‐period patients (*n* = 171) were excluded from comparative analyses. Virus positivity, coinfection rates, and demographic differences were assessed using chi‐square tests and *t*‐tests. Time‐series analysis using seasonal autoregressive integrated moving average models (SARIMA and SARIMAX) was conducted to compare observed and predicted trends.

**Results:**

Among 12,306 hospitalized children, 86.3% were in the prepandemic period and 12.3% in the postpandemic period. Median age rose from 20.0 to 27.0 months. Rhinovirus (21.3%–43.7%) and human metapneumovirus (6.6%–9.6%) increased, while influenza declined (4.8%–2.8%). Seasonal patterns shifted, with higher positivity in summer and autumn. Observed trends diverged from SARIMAX predictions, suggesting changes not fully explained by the meteorological variables included in the model. Coinfections with three or more viruses rose (1.7%–3.0%), and virus‐negative proportions decreased (32.6%–16.7%). RSV showed three peaks in the postpandemic period, suggesting possible shifts in viral cocirculation patterns.

**Conclusion:**

Shifts in pediatric respiratory virus patterns were observed in the postpandemic period and were not fully explained by meteorological variables alone, highlighting the need for adaptive surveillance incorporating broader environmental and demographic factors.

## 1. Introduction

The COVID‐19 pandemic has led to extensive research on pediatric respiratory infections, prompting a reassessment of their epidemiology and clinical impact [1–4]. Key infections include respiratory syncytial virus (RSV), rhinovirus, adenovirus, human parainfluenza virus, human metapneumovirus, and influenza, all of which contribute to acute lower respiratory tract infections in children and have shown altered incidence, transmission, and clinical effects [5–7]. The pandemic has significantly influenced respiratory virus activity, with studies like Chow et al. [1] highlighting the effects of public health interventions on virus trends.

RSV, a major cause of pediatric respiratory hospitalizations, saw its winter seasonality disrupted by nonpharmaceutical interventions (NPIs). While these measures curbed COVID‐19, they also reduced RSV circulation, delaying its seasonal peaks [8–11]. Similarly, human metapneumovirus, typically peaking from late winter to spring, displayed unexpected epidemiological shifts. The pandemic led to a sharp decline in overall respiratory virus infections, followed by a resurgence with altered seasonal patterns and increased coinfections [12, 13].

In the postpandemic period, respiratory viruses have not returned to prepandemic period patterns but, instead, show off‐season surges and increased coinfection rates, suggesting shifts in community immunity and virus–host interactions [1]. This phenomenon extends beyond an immunologic deficit, highlighting the complexity of respiratory virus epidemiology and the need for continuous global surveillance [1, 6, 8, 10, 14–17]. However, these studies primarily focused on specific viruses (e.g., RSV and human metapneumovirus) [10, 12, 13, 16, 17] or did not sufficiently address environmental factors, such as regional weather conditions, which may also influence the prevalence and severity of respiratory viral infections in the postpandemic period [17, 18].

In the Seoul metropolitan area, where the average annual temperature is 10°C–16°C and most rainfall occurs during the Changma (monsoon) season from late June to August, the unique climatic conditions warrant region‐specific investigation [19]. Recognizing that temperature, humidity, and virus transmission dynamics vary by location, we applied multivariate Seasonal Autoregressive Integrated Moving Average with Exogenous Regressors (SARIMAX) models incorporating meteorological variables to enhance prediction accuracy. Using data from 2012 to 2023, we aimed to map the distinct postpandemic trajectories of these respiratory viruses.

## 2. Methods

### 2.1. Study Design and Data Collection

This retrospective study analyzed pediatric respiratory virus infection data collected from January 2012 to December 2023, encompassing prepandemic, pandemic, and postpandemic periods, to assess the epidemiological and clinical impacts of COVID‐19 on respiratory virus infections among hospitalized children. Over the 12‐year period, anonymized data from hospitalized children who underwent respiratory virus testing at a tertiary hospital were collected from the medical laboratory records and analyzed [6, 20]. We included children hospitalized with respiratory tract infections, including lower respiratory tract infections (bronchitis, pneumonia, and bronchiolitis) and conditions with overlapping viral etiology, such as croup and asthma exacerbations. Croup, although primarily involving the larynx and trachea, shares viral pathogens with lower respiratory tract infections and has been included in prior pediatric respiratory surveillance studies. Asthma exacerbations in children are predominantly triggered by viral respiratory infections and were included to capture the full spectrum of virus‐associated respiratory morbidity requiring hospitalization. This study was approved by the Institutional Review Board (IRB) of CHA Bundang Medical Center (no. 2022‐08‐020). The requirement for informed consent was waived by the IRB because we used deidentified individual data for research purposes. Reporting follows the STROBE guidelines for observational studies (see Table S1).

### 2.2. Detection of Respiratory Pathogens Using Multiplex PCR Panels

We selected major respiratory viruses (RSV, rhinovirus, adenovirus, human parainfluenza virus, human metapneumovirus, and influenza) based on their prevalence and impact [21]. Cases included in this study met the following criteria: (1) hospitalization with a primary diagnosis related to respiratory tract infection and (2) respiratory virus testing performed using real‐time RT‐PCR or RT‐PCR methods during the hospitalization period. Cases were included regardless of test positivity; virus‐negative cases (30.8%) were retained in the analysis as a reference group. Virus detection used the Seeplex RV 7 Detection Kit (Seegene Inc.) from January 2012 to September 2015, the Anyplex II RV16 Detection Kit (Seegene Inc.) from October 2015 to June 2019, and the RV Multiplex 16 Detection Kit (LG463, Seegene Inc.) from July 2019 onwards. Although different multiplex PCR panels were used during the study period, all six target viruses were included across all three panels, and prior comparative studies have demonstrated comparable sensitivity across Seegene platforms for these target viruses [22, 23]. Assays followed manufacturer instructions and used undiluted nasopharyngeal swab specimens. For the comparative analysis between pre‐ and postpandemic periods, patients hospitalized during the pandemic period (February 2020 to November 2021, *n* = 171) were excluded, as this period was characterized by atypical suppression of respiratory virus circulation due to nonpharmaceutical interventions, including social distancing and school closures [2]. The postpandemic period was defined as beginning in December 2021, corresponding to the documented reemergence of respiratory virus circulation in Korean children from late 2021 onwards [24]. Accordingly, the comparative analysis included 10,621 prepandemic period cases (January 2012 to January 2020) and 1514 postpandemic period cases (December 2021 to December 2023).

### 2.3. Covariates and Outcome Variables

Patient data were extracted from electronic health records (EHRs) and included demographic and clinical information such as age, sex, admission date, and clinical diagnosis. Age was used to characterize demographic differences in infection patterns across study periods rather than as a covariate in the predictive models. In addition, data on incidence rates, seasonal distribution, and coinfection rates were obtained from the hospital′s microbiology database. Meteorological data, including temperature and humidity, were sourced from the Korea Meteorological Administration [19] and further supplemented by climate data collected from an automatic weather station (AWS) in Seoul (37.57 N, 126.97 E).

### 2.4. Statistical Analysis

Univariate regression analyses were conducted to evaluate the association between virus detection and each meteorological variable. Descriptive statistics were used to summarize the demographic and clinical characteristics of the study population. Virus positivity rates for the six representative respiratory viruses were measured and compared between the prepandemic and postpandemic periods. Seasonal patterns and coinfection rates were analyzed using chi‐square tests for categorical variables and *t*‐tests for continuous variables, as appropriate. Statistical significance was set at *p* < 0.05.

### 2.5. Seasonal Autoregressive Integrated Moving Average (SARIMA) Model

Monthly virus positivity rates were calculated as the number of specimens positive for each virus divided by the number of specimens tested for the corresponding virus in the same month, multiplied by 100. Given the seasonal nature of the data, we employed a SARIMA model to analyze temporal trends in respiratory virus positivity rates [11]. Prior to model fitting, stationarity was assessed using the augmented Dickey–Fuller (ADF) test, with differencing applied where necessary to achieve stationarity. Box–Cox transformation was applied when necessary to stabilize variance, and model predictions were back‐transformed to the original percentage scale for interpretation and visualization. Model orders (*p*, *d*, *q*) × (*P*, *D*, *Q*)_12_ were optimized independently for each of the six target viruses, as the seasonal patterns and autocorrelation structures differed among viruses. Candidate models were identified using autocorrelation function (ACF) and partial autocorrelation function (PACF) plots, and the optimal model for each virus was selected based on the Akaike information criterion (AIC). Seasonal periodicity was fixed at *s* = 12 to reflect the monthly structure of the data. Two‐month moving averages were used for graphical presentation to reduce short‐term fluctuations, whereas model fitting and performance evaluation were conducted using the monthly positivity rates.

Building on the optimal SARIMA model, we developed an extended SARIMA model that incorporated meteorological factors as external regressors. This extended model is referred to as the SARIMAX model. External regressors included monthly mean temperature and relative humidity, sourced from the Korea Meteorological Administration. The equation of a SARIMAX (*p*, *d*, *q*) × (*P*, *D*, *Q*)*s* model for a time series *Y*_*t*_ was as follows:

ΦBφB121−Bd1−B12DYt=ΘBθB12εt+ΣβnXn,t,

where *Y*_*t*_ is the monthly virus positivity rate at time *t*; *φ*(*B*) and *Φ*(*B* ^12^) are the nonseasonal and seasonal autoregressive operators, respectively; *θ*(*B*) and *Θ*(*B* ^12^) are the nonseasonal and seasonal moving average operators; *d* and *D* denote the orders of nonseasonal and seasonal differencing, respectively; *X*_*n*,*t*_ represents the *n*th external regressor at time *t*; *β*_*n*_ is the corresponding regression coefficient; and *ε*_*t*_ is the white noise error term.

Model adequacy was assessed through residual diagnostics, including inspection of residual ACF/PACF plots to confirm the absence of remaining temporal dependence. The SARIMAX models were trained using prepandemic period data from January 2012 to December 2019 and testing sets for internal validation. Model performance was evaluated by comparing predicted and observed monthly positivity rates using root mean square error (RMSE) and mean absolute error (MAE). All analyses were conducted in R Version 4.0.2 (R Foundation for Statistical Computing, Vienna, Austria), with the forecast, tseries, stats, ggplot2, and dplyr packages.

## 3. Results

### 3.1. Study Population Characteristics

Over the 12‐year study period from 2012 to 2023, a total of 12,306 children were hospitalized with respiratory tract infections, averaging 1025 children per year. The mean age was 32.7 months (SD 34.4), and 55.8% of the patients were male. The characteristics of the study population are presented in Table 1.

**Table 1:** Clinical and demographic characteristics of the study subjects (*n* = 12,306).

Variables	Value (%)
Sex	
Male	6861 (55.8)
Female	5445 (44.2)
Virus	
RSV	3227 (26.2)
Rhinovirus	2928 (23.8)
Adenovirus	1704 (13.8)
Human parainfluenza virus	1359 (11.0)
Human metapneumovirus	848 (6.9)
Influenza	553 (4.5)
Coinfection	
Negative	3792 (30.8)
1 positive infection	6659 (54.1)
2 positive infections	1630 (13.2)
3 positive infections	201 (1.6)
4+ positive infections	24 (0.2)
Diagnosis	
Croup	773 (6.3)
Bronchitis	5817 (47.3)
Pneumonia	1406 (11.4)
Bronchiolitis	3209 (26.1)
Asthma	371 (3.0)
Other	730 (5.9)
Admission year	
Prepandemic period (Jan. 2012 to Jan. 2020)	10,621 (86.3)
Pandemic period (Feb. 2020 to Nov. 2021)	171 (1.4)
Postpandemic period (Dec. 2021 to Dec. 2023)	1514 (12.3)
Admission season	
Spring	3300 (26.8)
Summer	2032 (16.5)
Fall	3774 (30.7)
Winter	3200 (26.0)

Abbreviation: RSV, respiratory syncytial virus.

### 3.2. Changes in Respiratory Virus Positivity Rates and Diagnoses Before and After the COVID‐19 Pandemic

Table 2 presents a comparative analysis of subject characteristics, respiratory virus prevalence, and clinical diagnoses between the prepandemic and postpandemic periods. Of 12,306 cases, 10,621 were from the prepandemic period and 1514 from the postpandemic period. A significant shift in age distribution was observed, with infections more common in children under 23 months in the prepandemic period (54.6%), whereas in the postpandemic period, cases increased in those aged 24 months or older (52.8%). The mean age increased from 31.6 (SD 33.7) to 39.2 (SD 37.9) months (*p* < 0.001) and the median from 20.0 (8.0–43.0) to 27.0 (12.0–53.0) months. In the postpandemic period, rhinovirus cases rose from 21.3% to 43.7% (*p* < 0.001) and human metapneumovirus from 6.6% to 9.6% (*p* < 0.001), while influenza declined from 4.8% to 2.8% (*p* < 0.001). Coinfection rates increased from 14.2% to 23.2% (*p* < 0.001), and fall infections became more frequent (35.2% vs. 30.5%, *p* < 0.001). The distribution of diagnoses shifted in the postpandemic period, with bronchitis remaining the most common diagnosis in both periods (48.1% vs. 46.6%), while other diagnoses increased substantially from 3.8% to 10.0%.

**Table 2:** Comparison of respiratory virus infection rates and diagnoses during pre‐ and post–COVID‐19 pandemic periods.

	Number, %^a^		*p* value^b^
	Prepandemic period (*N* = 10,621)	Postpandemic period (*N* = 1514)	
Subjects			0.019
Male	5962 (56.1)	899 (59.4)	
Female	4659 (43.9)	615 (40.6)	
Common six viruses			
RSV			0.095
No	7824 (73.7)	1084 (71.6)	
Yes	2797 (26.3)	430 (28.4)	
Rhinovirus			< 0.001
No	8354 (78.7)	853 (56.3)	
Yes	2267 (21.3)	661 (43.7)	
Adenovirus			0.434
No	9140 (86.1)	1291 (85.3)	
Yes	1481 (13.9)	223 (14.7)	
Human parainfluenza virus			0.001
No	9469 (89.2)	1307 (86.3)	
Yes	1152 (10.8)	207 (13.7)	
Human metapneumovirus			< 0.001
No	9919 (93.4)	1368 (90.4)	
Yes	702 (6.6)	146 (9.6)	
Influenza			< 0.001
No	10,111 (95.2)	1471 (97.2)	
Yes	510 (4.8)	43 (2.8)	
Coinfection			< 0.001
None	3414 (32.1)	256 (16.9)	
Single	5703 (53.7)	907 (59.9)	
Coinfection	1504 (14.2)	351 (23.2)	
Season			< 0.001
Spring	2850 (26.8)	450 (29.7)	
Summer	1656 (15.6)	376 (24.8)	
Fall	3241 (30.5)	533 (35.2)	
Winter	2874 (27.1)	155 (10.2)	
Diagnosis			< 0.001
Croup	699 (6.6)	74 (4.9)	
Bronchitis	5112 (48.1)	705 (46.6)	
Pneumonia	1260 (11.9)	146 (9.6)	
Bronchiolitis	2804 (26.4)	405 (26.8)	
Asthma	338 (3.2)	33 (2.2)	
Other	408 (3.8)	151 (10.0)	
Age			
< 23 months	5795 (54.6)	715 (47.2)	< 0.001
≥ 24 months	4826 (45.4)	799 (52.8)	

Abbreviations: COVID‐19, coronavirus disease 2019; RSV, respiratory syncytial virus.

^a^Pandemic‐period cases (February 2020 to November 2021, *n* = 171) were excluded from this comparative analysis.

^b^ *p* values were recalculated by chi‐square test following adjustment of denominators.

### 3.3. Epidemiological Shifts in Respiratory Virus Seasonality: Pre‐ and Postpandemic Period Comparison (2012–2023)

**Figure 1:**
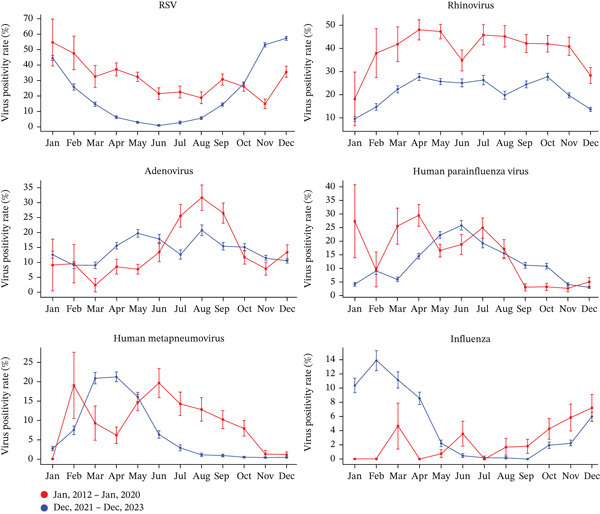
Monthly respiratory virus positivity rates in the prepandemic and postpandemic periods. The monthly virus positivity rate was calculated as the number of virus‐positive specimens divided by the total specimens tested for the same virus in the same month, multiplied by 100. Blue lines indicate the prepandemic period (January 2012 to January 2020), and red lines indicate the postpandemic period (December 2021 to December 2023). The *x*‐axis represents calendar month, the *y*‐axis represents positivity rate (%), and values are displayed by virus type.

Figure 1 shows monthly respiratory virus epidemic trends, comparing prepandemic and postpandemic periods. Seasonal virus positivity patterns changed significantly, with RSV shifting from a late autumn–winter peak in the prepandemic period to a more variable pattern, increasing from late summer to winter in the postpandemic period. Rhinovirus positivity remained consistently higher in the postpandemic period, especially in spring and autumn, while human metapneumovirus increased notably from winter to early summer. Influenza positivity declined markedly in the postpandemic period, whereas adenovirus and human parainfluenza virus exhibited greater variability without clear seasonal trends. These findings suggest substantial shifts in respiratory virus seasonality following the pandemic.

### 3.4. Modeling Versus Reality: Shifts in Seasonal Patterns of Respiratory Virus Positivity Rates in the Pre‐ and Postpandemic Periods

**Figure 2:**
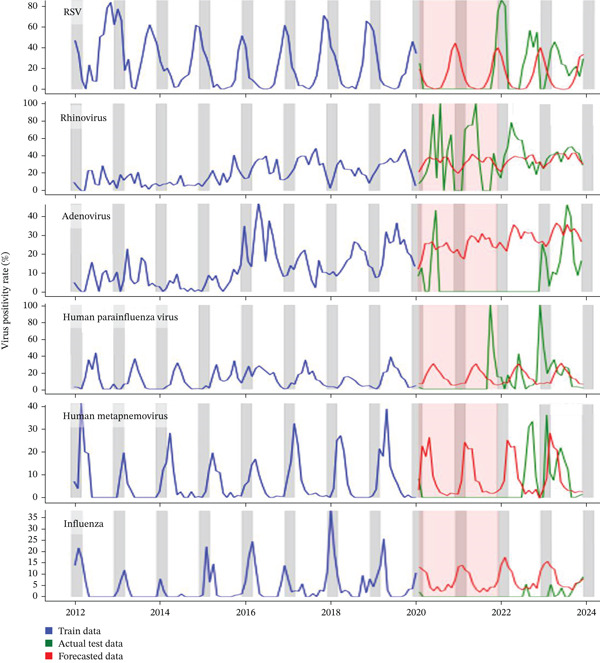
Observed and predicted monthly respiratory virus positivity rates, January 2012 to December 2024. Virus positivity rate was defined as the number of virus‐positive specimens divided by the number of specimens tested for the corresponding virus in the same month, multiplied by 100. Values are presented as 2‐month moving averages. The *x*‐axis indicates calendar time, and the *y*‐axis indicates virus positivity rate (%). The shaded region denotes the COVID‐19 pandemic period. Observed training‐period data, observed test‐period data, and model‐predicted values are shown separately. Predicted positivity rates were estimated using a SARIMAX model incorporating monthly mean temperature and relative humidity. Box–Cox transformation was applied to stabilize variance before model fitting, and model predictions were back‐transformed to the original positivity‐rate scale for visualization.

Figure 2 shows significant shifts in respiratory virus seasonality, comparing data from the prepandemic period with the postpandemic period. During the prepandemic period, seasonal peaks were well defined (e.g., RSV in winter), whereas in the postpandemic period, positivity rates increased during atypical months like summer and autumn. Using the SARIMAX model, we predicted cases based on prepandemic period trends. However, actual postpandemic period data exhibited irregular fluctuations, deviating from projections. These findings indicate that the COVID‐19 pandemic had a substantial impact on respiratory virus transmission patterns.

### 3.5. Viral Coinfection Rates

**Figure 3:**
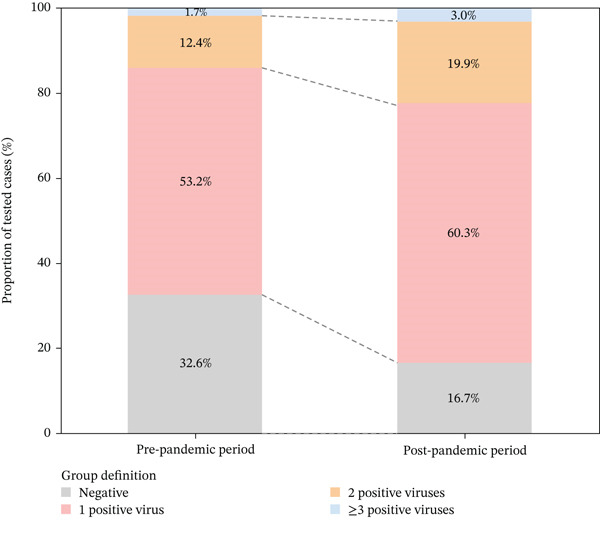
Comparison of respiratory virus coinfection rates between the prepandemic and postpandemic periods. Stacked bars show the distribution of respiratory virus test result categories during the prepandemic and postpandemic periods. Percentages were calculated using the total number of tested cases within each period as the denominator. Test results were categorized as negative, one positive virus, two positive viruses, or three or more positive viruses. The *x*‐axis represents the two study periods, and the *y*‐axis represents the proportion of tested cases (%). Values inside the bars indicate the percentage of tested cases in each category. Dashed lines connect corresponding category boundaries between periods to illustrate changes in category proportions. Differences in the distribution of test result categories between the prepandemic and postpandemic periods were assessed using the chi‐square test.

Figure 3 compares virus coinfection rates during the prepandemic and postpandemic periods. In the prepandemic period, 32.1% of cases tested negative for any virus, 53.7% had 1 positive result, and 14.2% exhibited 2 positive results. Only 1.7% of cases showed 3 or more virus infections. In the postpandemic period, the percentage of negative cases dropped to 16.9%, reflecting a reduction in virus‐negative results. The proportion of cases with 1 positive result increased to 59.9%, and those with 2 positive infections rose to 23.2%. Additionally, cases with 3 or more infections increased from 1.7% to 3.0%, suggesting a rise in coinfections.

This comparison indicates a shift in virus positivity rates, with fewer negative results and more coinfections during the postpandemic period. These findings may reflect changes in virus transmission dynamics or diagnostic practices following the COVID‐19 pandemic.

### 3.6. Respiratory Virus Positivity Rates by Age at Infection

**Figure 4:**
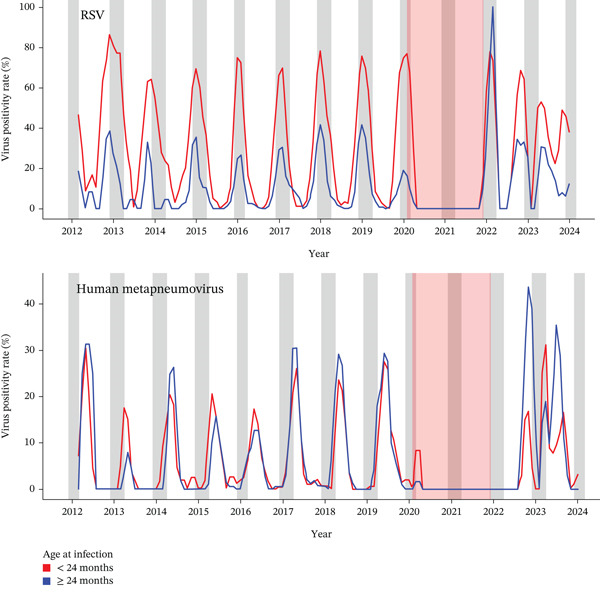
Monthly respiratory virus positivity rates by age group, January 2012 to December 2023 (2‐month moving average). The monthly virus positivity rate was calculated as the number of virus‐positive specimens divided by the total specimens tested for the same virus in the same month, multiplied by 100. Red lines indicate children under 24 months of age, and blue lines indicate children aged 24 months and older. The *x*‐axis represents calendar time, and the *y*‐axis represents positivity rate (%). In the prepandemic period, RSV positivity was predominantly observed in children under 24 months; in the postpandemic period, rates increased substantially in older children. A similar pattern was observed for human metapneumovirus.

The differences in age at infection between the prepandemic and postpandemic periods were analyzed. For RSV and human metapneumovirus, notable variations were observed. During the prepandemic period, RSV infections were primarily observed in children under 24 months of age. However, in the postpandemic period, a substantial increase in infection rates was noted among children aged 24 months and older. For human metapneumovirus, the age distribution of infections was similar in the prepandemic period. In the postpandemic period, however, a significantly higher positivity rate was observed in children aged 24 months and older (Figure 4).

### 3.7. Postpandemic Seasonality of Influenza and RSV

We speculated that the infection rates of these respiratory viruses might be interrelated and, therefore, examined the relationship between influenza and RSV, two viruses that exhibited marked changes in infection rates. Figure S1 illustrates the positivity rates of influenza (top panel) and RSV (bottom panel) from 2012 to 2023. For influenza, a significant reduction in positivity rates was observed throughout the pandemic period, and this declining trend persisted into the postpandemic period. In contrast, RSV exhibited notable seasonal shifts in the postpandemic period, with distinct peaks occurring in October 2022 and between March and April 2023.

## 4. Discussion

Our study results indicate a significant postpandemic shift in respiratory virus seasonality, with increased cases in summer and autumn. This deviation from traditional winter peaks underscores the pandemic′s impact on virus transmission. Higher infection rates among children aged 24 months and older compared to those under 24 months suggest changes in susceptibility, potentially due to reduced early‐life viral exposures during pandemic public health measures.

These findings align with global observations of off‐season epidemics and underscore the importance of continuous surveillance and adaptable public health strategies [3, 18]. Another study addressed potential factors driving the RSV rebound during the COVID‐19 pandemic, which might offer insights into the dynamics we have observed regarding increased infection rates among children aged 24 months and older in the postpandemic period [16]. The rise in dual and multiple viral infections in the postpandemic period suggests a complex interplay in virus interactions, reinforcing the importance of a multifaceted approach in future epidemiological studies. Similar patterns were reported in other regions, where changes in viral infection dynamics, including coinfections, were observed during the pandemic [15, 18].

Our predictive analysis, comparing modeled and actual respiratory virus positivity rates, demonstrates the impact of the COVID‐19 pandemic on virus transmission dynamics. While the model, based on prepandemic data, projected steady seasonal trends, postpandemic data revealed erratic fluctuations. This discrepancy highlights the need to recalibrate predictive models to account for unprecedented factors like a global pandemic. Incorporating weather variables such as temperature and humidity as external regressors offers insights beyond traditional predictions. Age‐related shifts in infection patterns, particularly the postpandemic increase in RSV and human metapneumovirus positivity among children aged 24 months and older, further underscore the importance of considering demographic factors in future epidemiological modeling. This divergence between modeled predictions and observed reality underscores the substantial changes in virus transmission dynamics induced by the pandemic, further emphasizing the importance of considering broader environmental and demographic factors in epidemiological modeling.

This study has several limitations. First, its retrospective design prevents establishing causality between respiratory virus epidemiology changes and pandemic‐era interventions. Unaccounted confounders such as public health policies and healthcare‐seeking behavior may further influence viral transmission beyond the meteorological covariates incorporated in the predictive models, and age was examined separately as a descriptive variable rather than a model covariate. Second, reliance on data from a single university hospital and national surveillance limits generalizability across regions with different healthcare policies, climates, or demographics. Third, the change in multiplex PCR panels in October 2015 represents a potential source of bias, particularly for coinfection rates, as broader panels may detect a greater number of simultaneous viral infections. However, all six target viruses were included in both the earlier and later panels, and prior comparative studies have demonstrated comparable sensitivity across these platforms for the target viruses used in this study [22, 23]. As the panel transition occurred within the prepandemic period, its impact on the primary pre‐ versus postpandemic comparison is likely limited, though residual influence on coinfection trends cannot be fully excluded. Fourth, predictive models may not fully capture the unprecedented disruptions to viral transmission dynamics caused by the COVID‐19 pandemic, limiting the accuracy of postpandemic trend forecasts. Fifth, the inclusion of croup and asthma exacerbations, conditions not conventionally classified as lower respiratory tract infections, may have influenced virus distribution estimates; although these diagnoses accounted for only 9.3% of the cohort, this potential influence cannot be entirely excluded. Sixth, the boundaries between study periods involve inherent uncertainty, as the definition of the postpandemic period relies on public health policy milestones rather than a virologically defined transition point. Future studies should prospectively define these boundaries and assess the robustness of findings to alternative period definitions.

This study adds to the expanding body of global evidence on the epidemiology of respiratory viruses in the post‐COVID‐19 era and highlights the importance of including climatic factors and age demographics in predictive models. Two key findings emerged. First, respiratory virus seasonality shifted, with increased incidence in summer and autumn, deviating from traditional winter peaks. Although temperature and humidity were incorporated as covariates in the predictive models, the extent to which climatic factors drove these shifts remains uncertain, and additional meteorological and behavioral variables should be considered in future studies. Second, age‐related susceptibility changed across the pandemic period, likely reflecting the combined influence of reduced early‐life viral exposures and relaxation of public health measures, though the underlying mechanisms warrant further investigation.

## 5. Conclusion

Postpandemic shifts in pediatric respiratory virus patterns, including increased rhinovirus and human metapneumovirus positivity, altered seasonality, and higher coinfection rates, were observed. These changes were not fully explained by meteorological variables alone, suggesting additional contributions from immunologic and behavioral factors. Adaptive surveillance strategies incorporating broader environmental and demographic variables are warranted.

## Funding

This study was funded by the Korea Health Industry Development Institute, 10.13039/501100003710, HR22C1605.

## Disclosure

The authors reviewed and edited the content as needed and take full responsibility for the content of the publication.

## Conflicts of Interest

The authors declare no conflicts of interest.

## Supporting information


**Supporting Information** Additional supporting information can be found online in the Supporting Information section. Table S1: STROBE statement. Figure S1: Observed and predicted monthly positivity rates for influenza and RSV, January 2012 to December 2023.

## Data Availability

The data that support the findings of this study are available from the corresponding authors upon reasonable request.
